# Cellulose Nanofiber Aerogel from Banana Peduncle Modified with Graphene Oxide as Bio-Adsorbent for Lead and Chromium Ions

**DOI:** 10.3390/gels11020095

**Published:** 2025-01-28

**Authors:** Anjar Priyatmojo, Riza Wirawan, Husaini Ardy, Dita Puspitasari, Putri P. P. Asri, Lia A. T. W. Asri

**Affiliations:** 1 Doctoral Program of Materials Science and Engineering, Faculty of Mechanical and Aerospace Engineering, Institut Teknologi Bandung, Jalan Ganesha 10, Bandung 40132, Indonesia; 33723001@mahasiswa.itb.ac.id; 2 Materials Science and Engineering Research Group, Faculty of Mechanical and Aerospace Engineering, Institut Teknologi Bandung, Jalan Ganesha 10, Bandung 40132, Indonesia; 3 Department of Biomedical Engineering, School of Electrical Engineering, Telkom University, Jalan Telekomunikasi 1, Bandung 40257, Indonesia

**Keywords:** heavy metal, adsorption, cellulose nanofiber, graphene oxide, aerogel

## Abstract

Textile industry waste contains high concentrations of heavy metals such as Pb(II) and Cr(VI) that must be reduced before they are released to the environment. The adsorption method is one way to reduce the heavy metal content. In this work, we develop a porous cellulose nanofiber (CNF) aerogel modified with graphene oxide (GO) as an alternative aerogel adsorbent for Pb(II) and Cr(VI). Cellulose was extracted from banana peduncle, a biomass waste that remains largely underutilized. The addition of GO aims to increase the adsorption properties. The aerogel adsorbents were synthesized by varying the ultrasonication time to 45 min for CNF 45 and 60 min for CNF 60, and the amount of GO added to 1 mL and 2 mL. The aerogel adsorbents were successfully prepared using the freeze-drying method with CNF45, CNF60, CNF45/GO1, CNF45/GO2, CNF60/GO1, and CNF60/GO2 variations. CNF was successfully isolated from a banana peduncle with an average diameter of 44.16 nm for 45 min (CNF 45) and an average diameter of 14.6 nm for 60 min (CNF 60) of ultrasonication. Chemical treatment and ultrasonication reduced the crystallinity index value of cellulose by 73% and 61% for CNF 45 and CNF 60, respectively. CNF aerogel has a very low shrinkage rate (<7%), resulting in a larger surface area. CNF60/GO2 obtained the optimum adsorption ability for Pb(II) metal at a concentration of 100 ppm and 27.27 mg/g at 30 min. On the other hand, the adsorption ability of Cr(VI) metal was obtained by CNF60/GO2 at a concentration of 100 ppm and 13.48 mg/g at 30 min. SEM images show that all aerogel adsorbents are porous, with a porosity value range of 96–98%. In conclusion, CNF60/GO2 proved to be the most effective aerogel adsorbent, offering the potential for heavy metal removal from industrial wastewater.

## 1. Introduction

The textile industry produces waste containing various heavy metals such as arsenic (As), cadmium (Cd), chromium (Cr)(VI), lead (Pb)(II), copper (Cu), and Zinc (Zn), with particularly high concentrations of Pb(II) and Cr(VI) found in waste streams, primarily originating from the dyes used in the manufacturing process [1]. The heavy metal Pb(II) and Cr(VI) content in waste must be below the threshold value of 0.05 mg/L [2]. Heavy metals exceeding the threshold are toxic, especially once they enter water, contaminating aquatic biotas and with no exception for humans. Organisms do not degrade heavy metals in the environment, which causes several health problems. Therefore, reducing or eliminating heavy metals from polluted water is necessary because they are at risk of accumulating in living organisms. Various methods for removing heavy metal ions from wastewater have been developed, such as coagulation [3], adsorption [4], filtration [5], and ion exchange [6]. Among these, adsorption is considered one of the most promising due to its high efficiency, cost-effectiveness, environmental friendliness, and ease of use [7]. This method relies on an adsorbent material capable of effectively binding heavy metals.

Cellulose is one of the most promising adsorbent candidates [8]. It can be obtained from agricultural biomass residues with several advantages, including low cost, environmental friendliness, abundance, reliable natural sources, suitable adsorption properties, and renewable properties [9]. Due to their high adsorption ability, agricultural waste materials containing cellulose have great potential as effective adsorbents for various pollutants [10]. Many studies have highlighted the use of biomass residues as sources of cellulose for adsorbent, specifically from coconut shells [11], banana peels [12], and pineapple leaves [13]. Bananas are the second most produced globally, representing 16% of the world’s total fruit production. The high levels of production and consumption lead to substantial waste, estimated to be 30–40% of the total fruit weight, resulting in around 3.5 million tons of waste annually. Previous researchers have explored banana peel [14], banana pith [15], and banana stem [16] as a cellulose source for the removal of heavy metals from contaminated waters. Among these, banana peduncles have not been widely utilized as a source of cellulose as an adsorbent. They contain 48.31–60.41% cellulose, making them a suitable cellulose-based adsorbent precursor because their hydroxyl group plays a role in binding heavy metal ions [17,18]. The cellulose-based adsorbent has several weaknesses, such as poor hydrophilicity and low chemical and physical stability, resulting in an insufficient adsorption capacity [19]. These weaknesses can be overcome by converting the cellulose to a nano-sized material to increase its adsorption ability because it has a high surface area, mechanical strength, and low density [20]. The size dimensions of cellulose nanofiber (CNF) can determine its surface area and the number of high hydroxyl groups, so the surface is easy to modify. CNF can be fabricated into aerogel, a three-dimensional (3D) porous solid characterized by an ultralow density, large surface area, and interconnected pore network. Aerogel is a type of special porous material typically derived from gels in which the liquid component is replaced with gas, resulting in a solid with excellent physical and chemical properties, such as low density (0.0005–0.35 g/cm^3^), high porosity (84–99.9%), large specific surface area (10–975 m^2^/g), and high compressive strength (5.2 kPa–16.67 MPa) [21]. Aerogel is considered a promising candidate for adsorbent use in heavy metal wastewater treatment due to its high specific surface area and tunable functionalization for selective adsorption [22].

Modification of CNF aerogel is required to enhance the adsorption of heavy metal ions. CNF aerogel can be effectively modified with carbon nanomaterials, such as graphene [16], graphene oxide (GO) [17], and reduced graphene oxide (rGO) [18], to improve its ability to adsorb heavy metal ions due to the increased active functional groups provided and to improve the mechanical properties of the aerogel adsorbent. Among these carbon nanomaterials, GO is one with a high surface area, which provides more active sites for chemical reactions, thus increasing its adsorption capacity [23].

The oxygen-containing functional groups on GO contribute significantly to its hydrophilicity and high negative charge density, which are important for heavy metal removal [24]. Kong et al. [25] prepared a graphene oxide/chitosan (GO/CS) composite aerogel that was synthesized using the freeze-drying method. The adsorbent has a good adsorption performance for Cr(VI), with the maximum adsorption amount being as high as 146 mg/g. Wei et al. successfully designed a nanocellulose–Fe_3_O_4_ hybrid aerogel with a high porosity of over 99% for the adsorption of heavy metal ions from water; the removal rate of adsorbent for Cr(VI) was (2.2 mg/g) and Pb(II) (1.25 mg/g) [26]. Furthermore, Jian Li et al. [27] prepared an aerogel adsorbent based on nanocellulose fiber (CNF) and polyethyleneimine (PEI) via electrostatic combination without chemical crosslinking. The achieved adsorption efficiency for Pb(II) was rapid and more than 90%. The CNF aerogel has been utilized in many fields, like organic dye adsorption [26], heavy metal adsorption [27,28], and oil adsorption [28,29].

Recently, several studies have explored aerogels made from nanocellulose and GO sheets for wastewater treatment. In our research, we utilized banana peduncle as an alternative source for CNF aerogel-based aerogel adsorbents, which has not been previously reported. We incorporated GO into the CNF aerogel to enhance its adsorption capacity and examined its effectiveness in adsorbing Pb(II) and Cr(VI) metal ions.

## 2. Results and Discussion

### 2.1. Preparation of CNF

**Figure 1:**
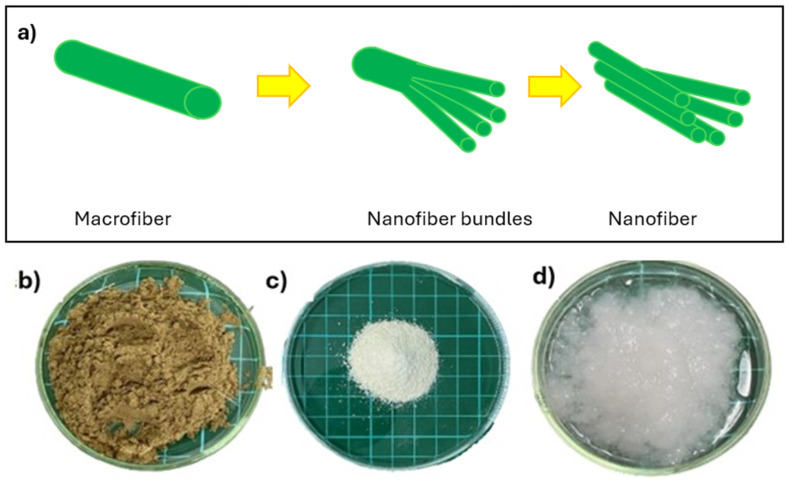
(**a**) Mechanism of cellulose nanofiber formation, (**b**) banana peduncle powder, (**c**) cellulose, (**d**) CNF.

Cellulose extraction from banana peduncles was the initial stage of this research, conducted to obtain pure cellulose before the CNF isolation. Pure cellulose was obtained from the powder of banana peduncles through an alkaline hydrolysis and bleaching process [28]. The dried banana peduncle fibers undergo alkaline hydrolysis to remove hemicellulose and lignin. The remaining lignin is then removed by bleaching in sodium hypochlorite (NaOCl) solution, which is capable of oxidizing the chromophoric groups present in lignin, resulting in pure white cellulose, as seen in Figure 1c. Bleaching with NaOCl can also dissolve lignin and other extractive substances [29]. The CNF was then prepared using an ultrasonication process. The ultrasonic waves create a cavitation effect, capable of breaking the secondary bonds in macro fiber strands into nanofibers, as illustrated in Figure 1a [30]. During the 60 min ultrasonication, cellulose nanofiber with a higher gel-like consistency was obtained compared to the 45 min ultrasonication, as observed in Figure 1d.

### 2.2. Characterization of CNF

**Figure 2:**
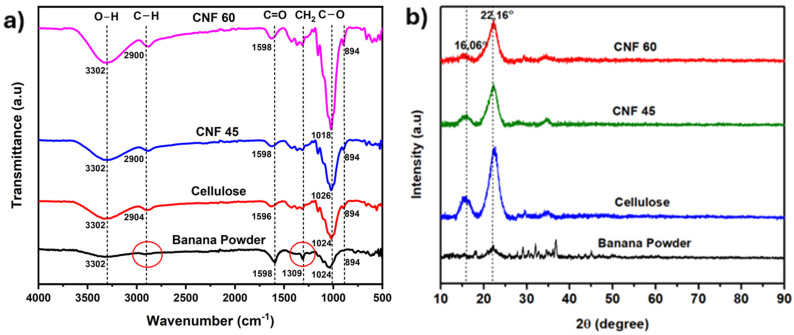
(**a**) FTIR spectrum of banana peduncle powder, cellulose, CNF 45, and CNF 60 (**b**) XRD diffractogram of banana peduncle powder, cellulose, CNF 45, and CNF 60.

Figure 2 displays the FTIR spectra of banana peduncle powder and cellulose powder, both showing a broad peak located in the range of 3450–3300 cm^−1^. This peak represents O–H bond stretching. The intensity of the –OH peak in the cellulose spectrum is higher than that of the banana powder after normalization. This indicates that the content of hydroxyl groups is higher in the cellulose sample compared to the banana powder sample, which contains other components such as lignin, hemicellulose starch, and other organic compounds that may have fewer hydroxyl groups. Additionally, the FTIR peak is enhanced in cellulose powder due to the hydroxyl groups’ involvement in intra and inter-molecular hydrogen bonding [31]. In contrast, the interactions in banana peduncle powder are weaker or more variable due to other components that may disrupt or weaken these interactions.

The peaks at around 2900 cm^−1^ originate from the C–H stretching vibration in the CH_2_ of the CH_2_–OH group of cellulose [32]. The aliphatic C–H groups in the samples are indicated by peaks at 2916 cm^−1^ for banana peduncle powder and 2904 cm^−1^ for cellulose. The peak at 1598 cm^−1^ in the banana powder sample indicates the OH bending of adsorbed water [33,34]. In contrast, in the cellulose sample, this peak is less sharp. This shows that the banana powder sample has a more complex composition than the cellulose sample, which potentially has a lower hemicellulose and lignin concentration because the cellulose sample is more homogeneous and consists mainly of cellulose itself, so the resulting peak is wider.

The disappearance of the sharp peak at 1309 cm^−1^ in cellulose indicates the removal of lignin [35]. Meanwhile, the peak in the spectrum around 1024 cm^−1^ can be attributed to the stretching of CO and C–C groups within cellulose. The peaks in 896–891 cm^−1^ represent the characteristic structure of cellulose, indicating the presence of β-glycosidic bonds in cellulose, involving the bending of C–H and O–H bonds [36]. Leng et al. mentioned that peaks around 1429 cm^−1^, 1163 cm^−1^, 1111 cm^−1^, and 897 cm^−1^ indicate cellulose type I-β [37]. This FTIR analysis confirms the successful isolation of pure cellulose from banana peduncles through the alkaline hydrolysis and bleaching processes. Figure 2a shows the FTIR spectra of banana powder, cellulose, CNF 45, and CNF 60, and there was no significant difference among the three spectra. This indicates that the duration of the ultrasonication process does not significantly alter the chemical structure of cellulose, CNF 45, and CNF 60.

XRD was conducted to obtain information about the crystal structure and crystallinity of banana powder, pure cellulose, and CNF, as seen in Figure 2b. The XRD diffractograms of cellulose and CNF show two characteristic peaks at 2θ angles of 16.06° and 22.16°. These peaks correspond to cellulose type I β, representing the crystal lattice planes (110) and (200), respectively [38]. The high peak at an angle of 2θ around 16.05° and 22.05° is characteristic of cellulose structure [39]. The crystallinity of cellulose was calculated using methods based on peak area. The intensity-based method employs the Segal equation, whereas the area-based approach uses a Gaussian function, resulting in crystallinity with values of 86% for the cellulose sample, 73% for CNF 45, and 61% for CNF 60. The decrease in crystallinity from cellulose to CNF can be attributed to the cavitation effect generated during ultrasonication. The cavitation effect breaks the secondary bonds in the cellulose structure, and as the ultrasonication time increases, more secondary bonds are broken, resulting in a reduction in crystallinity [40]. Therefore, it can be concluded that pure cellulose was successfully obtained through alkaline hydrolysis and bleaching treatment.

**Figure 3:**
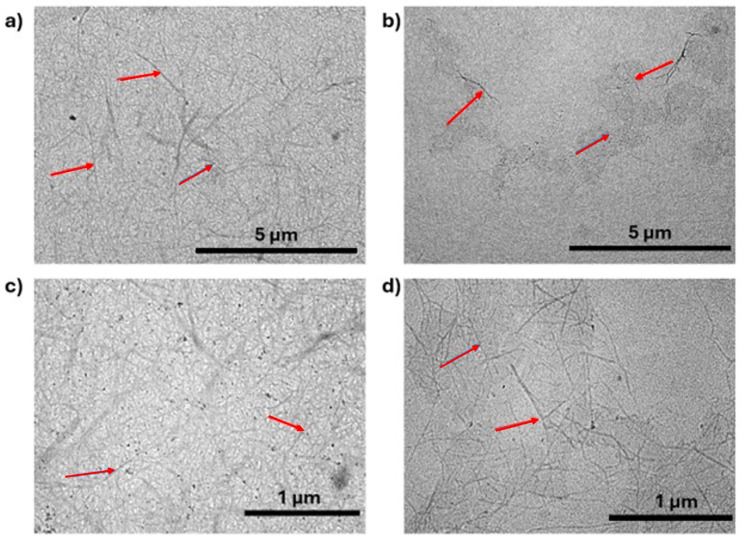

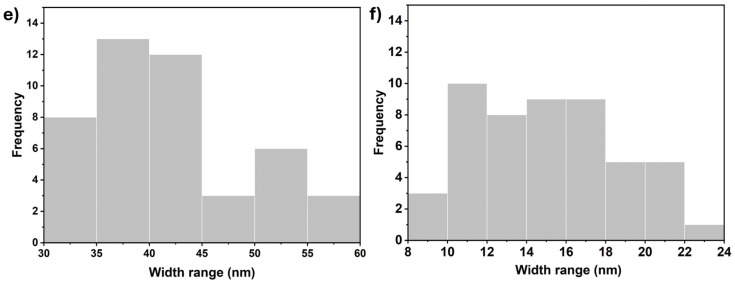
TEM images of (**a**,**c**) CNF 45 and (**b**,**d**) CNF 60, histogram of diameter distribution of (**e**) CNF 45 and (**f**) CNF 60, where show CNF indicated by arrows as shown in images a–d.

Banana peduncles produce CNF, shown by red arrows, and nanocrystalline cellulose (CNC), shown by blue arrows, through alkaline hydrolysis and bleaching treatment, as seen in the TEM images in Figure 3a–d. CNCs form primarily through the mechanical treatment of ultrasonication, which is a process that involves the application of high-frequency sound waves to break down cellulose fibers into smaller, more uniform particles. Both CNC and CNF are utilized in the fabrication of aerogels. However, CNF is generally more appropriate for most foam and aerogel applications because of its greater flexibility, fibrous structure, and capacity to form a robust 3D porous network upon dehydration [41].

The diameter size of CNF 45 ranges from 30 nm to 60 nm, with an average of 44.16 nm, while CNF 60 ranges from 8 nm to 24 nm, with an average of 14.6 nm. The CNF 45 aerogels have most diameters in ranges of 35–40 nm, while the CNF 60 ranges from 10 to 12 nm, as seen in the histogram in Figure 3e,f. It can be concluded that a longer ultrasonication time results in smaller CNF sizes. This occurs because ultrasonication generates cavitation energy, leading to fiber size changes. Ultrasonic waves cause the breakdown of macrofibril fibers due to the cavitation effect produced by shock waves in the medium. The ultrasonication process also erodes the fiber surface, allowing the fiber to split along the axial direction, gradually altering the structure and size of cellulose fibers [40,41]. Prolonged ultrasonication gives more opportunities for erosion and splitting, resulting in smaller and more uniform fibers [42]. Ultrasonication disrupts the intermolecular hydrogen bonds within cellulose, breaking down its crystalline regions into smaller, less ordered structures. The reduction in crystallinity correlates with the observed size decrease as fibers are further disintegrated into nanofibers [43]. The large difference in CNF diameters after 45 and 60 min of ultrasonication is a direct result of the accumulated cavitation energy, surface erosion, axial splitting, and disruption of hydrogen bonds over time. These combined effects effectively reduce the cellulose fiber size.

### 2.3. Fabrication of CNF and CNF-GO Aerogel Adsorbents

**Figure 4:**
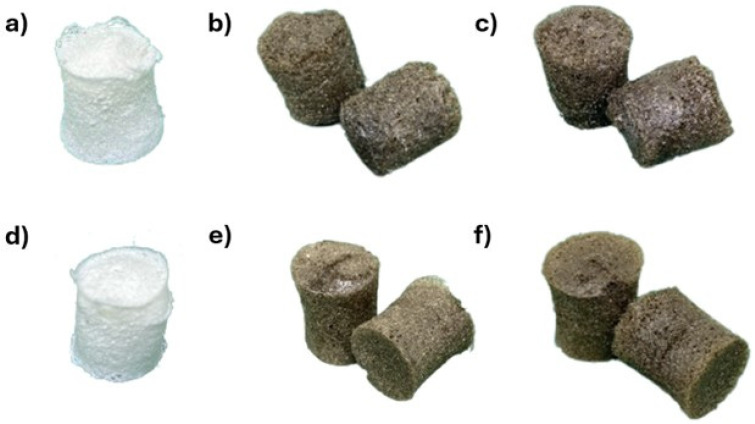
CNF 45 aerogel adsorbent (**a**), CNF 45 + 1 mL GO aerogel adsorbent (**b**), CNF 45 + 2 mL GO aerogel adsorbent (**c**), CNF 60 aerogel adsorbent (**d**), CNF 60 + 1 mL GO aerogel adsorbent (**e**), CNF 60 + 2 mL GO aerogel adsorbent (**f**).

The freeze-drying method was used to fabricate CNF and CNF-GO aerogel adsorbents. During freeze-drying, the water trapped inside a hydrogel will transform into ice crystals. Sublimation occurs when the ice crystals become voids or pores as the temperature decreases. CNF and CNF-GO aerogel adsorbents result in porous solid forms, and the addition of GO leads to a darker color change, as observed in Figure 4a–c.

**Figure 5:**
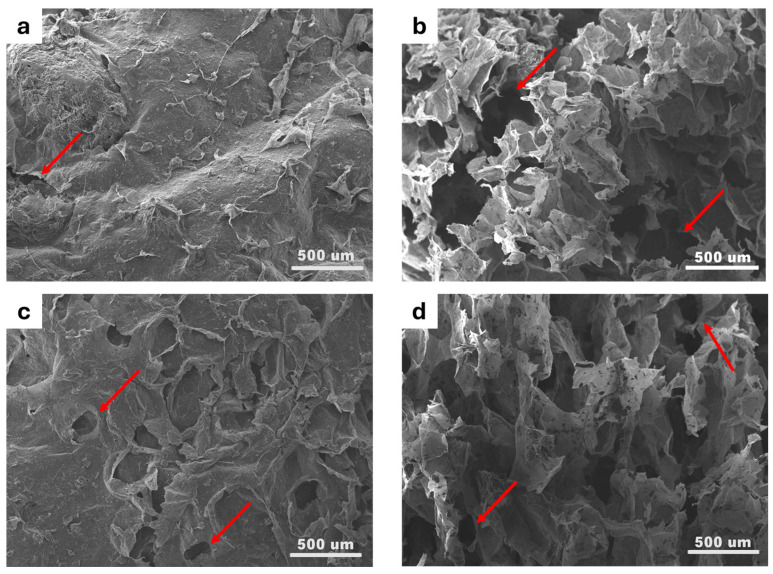

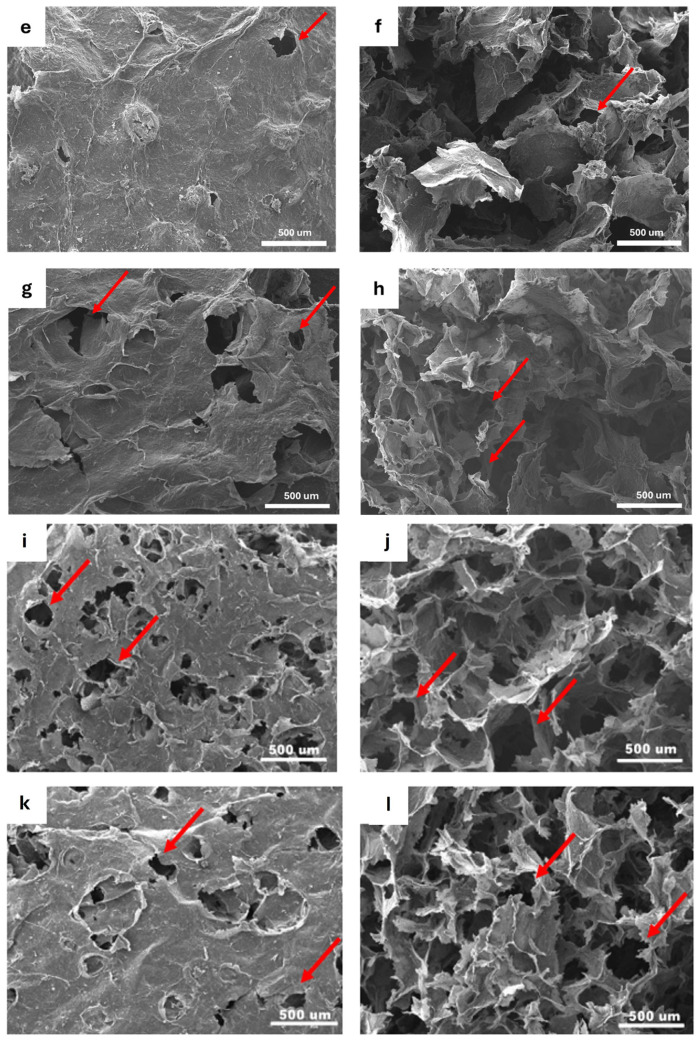
SEM images of surface of CNF 45 aerogel adsorbent (**a**), cross-section of CNF 45 aerogel adsorbent (**b**), surface of CNF 60 aerogel adsorbent (**c**), cross-section of CNF 60 aerogel adsorbent (**d**), surface of CNF 45 + 1 mL GO aerogel adsorbent (**e**), cross-section of CNF 45 +1 mL GO aerogel adsorbent (**f**), surface of CNF 45 + 2 mL GO aerogel adsorbent (**g**), cross-section of CNF 45 + 2 mL GO aerogel adsorbent (**h**), surface of CNF 60 + 1 mL GO aerogel adsorbent (**i**), cross-section of CNF 60 +1 mL GO aerogel adsorbent (**j**), surface of CNF 60 + 2 mL GO aerogel adsorbent (**k**), cross-section of CNF 60 + 2 mL GO aerogel adsorbent (**l**), where all images show pore indicated by arrows.

SEM images of the aerogel adsorbents subjected to ultrasonication for 45 and 60 min and the aerogel adsorbents ultrasonicated for 45 and 60 min mixed with 1 mL and 2 mL of GO (CNF + 1 mL GO and CNF + 2 mL GO) are shown in Figure 5a–h. All aerogel adsorbents are porous, as the red arrows show, but the resulting pore structures vary depending on the composition and treatment. SEM images of the surface of the CNF 45 and CNF 60 aerogel adsorbents show relatively fewer pores due to some shrinkage during the freeze-drying process. On the other hand, SEM images of the surface of the CNF aerogel adsorbents with added GO result in a more porous surface due to the inclusion of GO in the liquid suspension.

The SEM images exhibit more pore formation than the surface on the CNF 45 and CNF 60 aerogel adsorbent cross-sections. In contrast, the SEM images of the cross-sections of CNF aerogel adsorbents with added GO display irregular small pores with thin walls. In aerogel form, adding GO will create a three-dimensional network by producing many pores, thus increasing the surface area and porosity of CNF/GO aerogels [44]. However, in application, the addition of GO may reduce porosity due to the buildup of GO sheets. The following research was conducted by Nguyen et al. [45], who successfully produced CNF and GO aerogels with porous structures that vary depending on the composition of the material in the aerogel.

**Figure 6:**
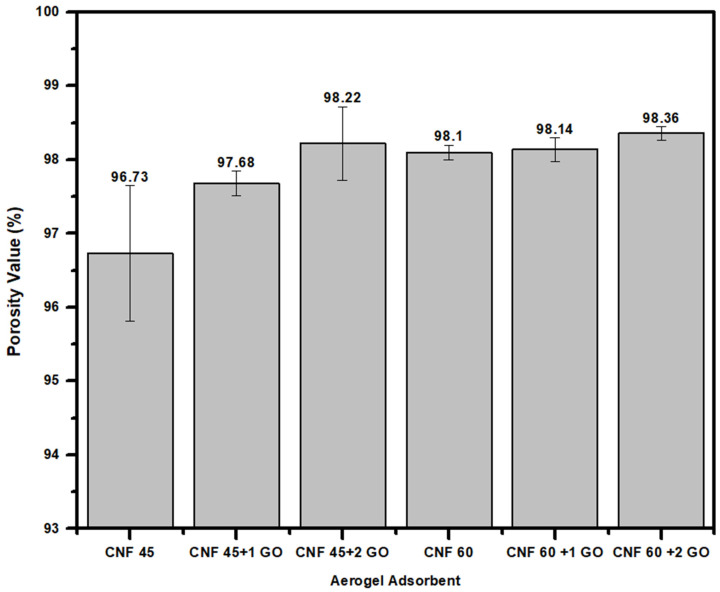
Porosity value results of aerogel adsorbents CNF and CNF/GO.

The porosity of CNF aerogel adsorbents and CNF-GO aerogel adsorbents was tested using butanol absorption. Figure 6 shows that the measured porosity value of the CNF 60 aerogel adsorbents of 98.1% is higher than for the CNF 45 aerogel adsorbent, which is only 96.73%. This is related to the fact that CNF produced after 60 min of ultrasonication will have smaller sizes compared to 45 min of ultrasonication, resulting in more pores. During ultrasonication, acoustic cavitation occurs, which involves the formation, growth, and collapse of microbubbles in the liquid medium. This process generates intense shear forces and microjets that can break down larger cellulose fibers into smaller nanofibers [46]. As the ultrasonication time increases, the energy input also increases, leading to more effective disintegration of the cellulose structure, resulting in smaller fiber sizes at 60 min compared to 45 min.

The relationship between ultrasonication time and the formation of many pores in the CNF aerogel structure occurs due to longer ultrasonication times, improving the dispersion of CNFs in aqueous media. A better dispersion will minimize agglomeration and allow for the formation of a more homogeneous structure so that the surface area and pore volume increase [47]. A smaller fiber size contributes to creating a network with a larger amount of interstitial space, which will increase the overall porosity of the material.

The addition of GO to both CNF 45 and CNF 60 aerogel adsorbents increases the porosity values. CNF aerogel adsorbents and CNF-GO aerogel adsorbents exhibit porosities in the range of 96–98%. This indicates that the freeze-drying process does not result in significant shrinkage. The porosity range falls within the aerogel porosity range, which is typically between 84 and 99.9% [21]. In this study, the CNF 45 + 2 mL GO aerogel achieved the highest porosity value of 98.22%, similar to that reported by Nguyen et al. [45], where CNF/GO aerogels had a maximum porosity value of 98.2%.

**Figure 7:**
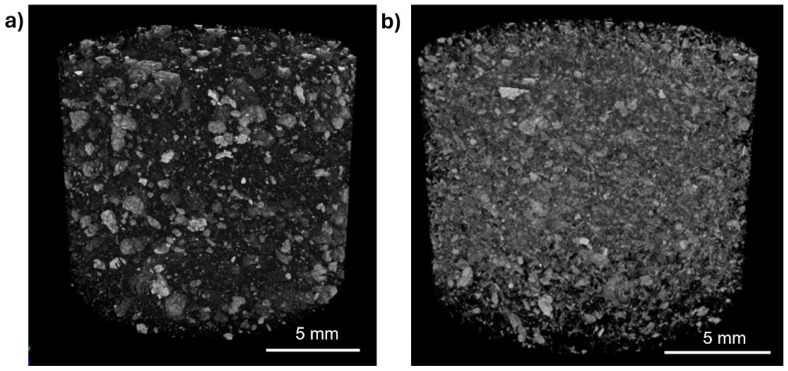
Micro-CT 3D image of (**a**) CNF 60 and (**b**) CNF 60 + 2 mL GO aerogel adsorbents with scale 5 mm.

The micro-CT test resulted in 1053 slice images for the CNF 60 aerogel adsorbents and 856 slice images for the CNF 60 + 2 mL GO aerogel adsorbents. The CTvox (version 3.3.0, Skyscan, Bruker, Billerica, MA, USA) data viewer software was used to convert the slice images into a 3D model by stacking and reconstructing them to obtain a picture, as shown in Figure 7a,b. The micro-CT test is a well-established technique to identify a material’s porosity without sectioning the sample [48]. In this study, the total porosity and the interconnected pore of the aerogel adsorbents are shown in Table 1. The sample presented high porosity, from 91% to 92%. The porosity value results are lower than the butanol method. This is because the micro-CT method has a resolution limit, usually in the micron range, which means it cannot detect very small pores that can be accessed by butanol. The sample CNF 60 aerogel adsorbents showed higher total porosity than the CNF 60 + 2 mL GO aerogel adsorbents. The added porosity of GO is smaller than that of pure CNF aerogel due to the over-stacking phenomenon of graphene sheets, which can lead to a loss of surface area during drying [49]. The addition of GO can reduce porosity as the buildup of GO sheets during the fabrication process can create dense regions within the aerogel, thus preventing the formation of pores [50].

Micro-CT analysis shows a lower porosity when compared to the butanol method because the butanol method has limitations in accurately characterizing pores due to the wide-scale range of void space. The micro-CT method, although having some advantages such as direct 3D analysis, time efficiency, and non-destructive imaging, has a weakness in the challenge of representing the actual specimen precisely because of the partial volume effect, the structure below the resolution limit, and the low X-ray density. Micro-CT images can also be influenced by computer tomography artifacts and image noise, which affect the calculation of certain pore size parameters [51]. However, no one method is considered good enough because different techniques can produce different porosity values according to the type of pore and its size range. These limitations highlight the need for careful consideration when choosing between butanol testing and micro-CT for porosity analysis based on the specific requirements of the study.

### 2.4. Pb(II) Adsorption

The adsorption test for CNF aerogel adsorbents and CNF-GO aerogel adsorbents was conducted by immersing them in Pb(II) solutions with 50 ppm and 100 ppm concentrations. The initial aim of the study was to evaluate the adsorption performance of CNF-based aerogels at two concentration ranges around 50 ppm (low concentration) and 100 ppm (high concentration) [52]. The measured concentrations of 104.4 ppm, 93.57 ppm, and 57.61 ppm were the resultant working concentrations after preparing the Pb(II) solutions and calibrating for experimental accuracy. These values closely align with the intended target ranges. The effect of contact time on the adsorption of Pb(II) varied between 15 and 30 min at around 100 ppm and at 15 min for around 50 ppm [53].

**Figure 8:**
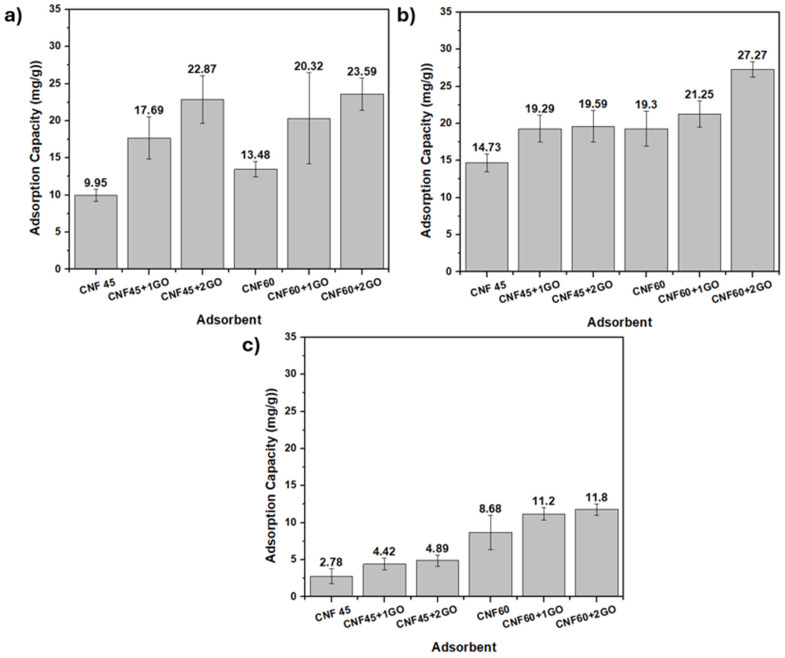
(**a**) Histogram of AAS analysis result in Pb(II) solution with a concentration of 104.4 ppm for 15 min, (**b**) 93.57 ppm for 30 min, (**c**) 57.61 ppm for 15 min.

The Atomic Absorption spectroscopy (AAS) analysis results for the Pb(II) solution with a control of 100 ppm Pb(II) metal showed a concentration of 104.4 ppm. The AAS testing results for the same solution with an adsorption contact time of 15 min can be seen in Figure 8a. The adsorption capacity of CNF 60 aerogel adsorbents is better than that of CNF 45 aerogel adsorbents. The adsorption capacity of CNF 60 aerogel adsorbents is 13.48 mg/g, while CNF 45 aerogel adsorbents have an adsorption capacity of 9.95 mg/g. This is related to the CNF produced with a 60 min ultrasonication time, resulting in a higher surface area than a 45 min ultrasonication time and thus having more pores.

The relationship between the porosity data and the adsorption capacity can be explained by the fact that higher porosity, as observed in the CNF 60 aerogel adsorbents, directly correlates with increased surface area. As mentioned in our porosity data in Figure 6, CNF 60 aerogel adsorbents, which underwent 60 min of ultrasonication, have smaller fiber sizes and more pores compared to CNF 45 aerogel adsorbents, leading to a higher porosity value. This increased porosity creates more available binding sites for the Pb(II) metal to interact with the OH groups present in cellulose, enhancing the adsorption capacity. Therefore, CNF 60, with a higher porosity and better dispersion, allows for more efficient Pb(II) adsorption than CNF 45. Additionally, Pb(II) metal can bind with the OH groups present in cellulose, so smaller CNF sizes will have a better bond with heavy metal Pb(II).

Adding GO to CNF 45 and CNF 60 aerogel adsorbents can enhance their adsorption capacity. In the CNF 45 aerogel adsorbents, there was an increase in adsorption capacity of 7–13 mg/g, while in the CNF 60 aerogel adsorbents, there was an increase in adsorption capacity of 7–10 mg/g. This indicates that GO can improve its ability to bind with Pb(II) metal through its functional groups. However, the addition of GO resulted in an increase of only 10–13 mg/g. This could be because GO is trapped within the CNF matrix, preventing direct contact with Pb(II) metal. The optimal adsorption capacity was achieved with CNF 60 aerogel adsorbents by adding 2 mL of GO, and was 23.59 mg/g.

The CNF 60 aerogel exhibits superior adsorption performance compared to other adsorbents due to its fine nanofiber diameter (14.6 nm), which provides a high surface area and abundant active sites for adsorption. The addition of 2 mL of GO further enhances this capacity because it improves the adsorbent’s surface area, porosity, and availability of functional groups, such as hydroxyl (-OH), carboxyl (-COOH), and epoxy (-C-O-C) groups, which strengthen the adsorbent’s affinity toward heavy metal ions, particularly Pb(II). These functional groups facilitate chemical interactions, such as complexation and electrostatic attraction, improving adsorption efficiency.

The addition of 2 mL GO optimizes the adsorbent’s performance by increasing the availability of adsorption sites while maintaining structural integrity. GO’s two-dimension (2D) nanosheet structure contributes significantly to the dispersibility and accessibility of functional groups, enabling efficient ion binding. Furthermore, GO’s synergistic interaction with CNF enhances the mechanical stability and porosity of the aerogel, resulting in a robust and efficient adsorbent [52,53,54].

The AAS analysis resulted in Pb(II) solutions with longer adsorption contact times, precisely 30 min, and a control metal Pb(II) concentration of 93.57 ppm, as seen in Figure 8b. Compared to a 15 min contact time, a 30 min contact time shows a higher adsorption capacity for the CNF aerogel adsorbents and CNF-GO aerogel adsorbents. However, there was no significant difference between the various CNF and GO variations. This is likely due to the aerogel adsorbents having reached saturation. The higher adsorption capacity is due to the variation in adsorption time. A longer adsorption time of 30 min provides more opportunities for interaction between the active sites of the aerogel adsorbent and Pb(II) ions. This extended interaction time increases the likelihood of achieving equilibrium adsorption, resulting in a higher adsorption capacity [55,56,57].

The adsorption mechanism involves both physical and chemical processes. If Pb(II) metal is not firmly bound to the OH groups on the aerogel adsorbent and other functional groups of GO, it will undergo physical adsorption. In this process, Pb(II) metal will undergo adsorption mechanisms of release and re-adsorption, repeatedly filling the pore space until reaching saturation.

The AAS analysis results for Pb(II) solutions with a control of 50 ppm Pb(II) metal showed a concentration of 57.61 ppm. The AAS testing results for this solution with a 15 min adsorption contact time can be seen in Figure 8c. At a concentration of 57.61 ppm and with a 15 min contact time, the results are similar to the 104.4 ppm concentration at a 15 min contact time. This means that the CNF-GO aerogel adsorbents have a better adsorption capacity than CNF. Incorporating GO can enhance adsorption capacity due to the increased availability of oxygen-containing functional groups for interacting with contaminants, additional pores and channels within the aerogel structure, and a significantly larger specific surface area for adsorption [58].

At a concentration of 57.61 ppm with a 15 min contact time, the results are consistent with those obtained at 104.4 ppm with a 15 min contact time. In both cases, the CNF-GO aerogel adsorbent demonstrates superior adsorption capability compared to the CNF aerogel adsorbent, and the adsorption capacity increases with the addition of GO volume. The optimum adsorption capacity is achieved with CNF 60 aerogel adsorbents by adding 2 mL of GO, corresponding to 27.27 mg/g.

Vargas et al. [59] reported that using CNC as a lead metal ion adsorbent gives an adsorption capacity of 17.9 mg/g. This study shows that using CNF as a lead metal ion adsorbent is more effective, with more than double the adsorption capacity. This effectiveness is due to the larger surface area and pore volume of CNF compared to CNC, which increases the adsorption capacity.

**Figure 9:**
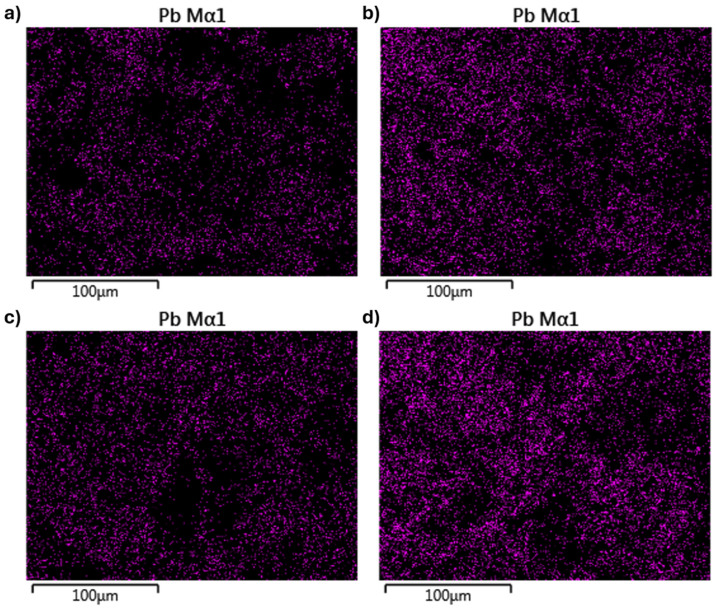
EDX mapping result of the distribution of Pb(II) on CNF 45 aerogel adsorbent (**a**), CNF 45 + 2 mL GO aerogel adsorbent (**b**), CNF 60 aerogel adsorbent (**c**), and CNF 60 + 2 mL GO aerogel adsorbent (**d**).

EDX mapping characterization was conducted on the aerogel adsorbents’ cross-section to confirm the Pb(II) distribution that bound to the aerogel adsorbents. CNF 45 aerogel adsorbents have a limited distribution compared to CNF 60 aerogel adsorbents. Meanwhile, the CNF 45 + 2 mL GO aerogel adsorbents have an even more limited distribution than CNF 60 + 2 mL GO aerogel adsorbents. The distribution of Pb(II) metal is displayed as purple-colored dots, as shown in Figure 9a–d. This follows the previously obtained data showing that the CNF aerogel adsorbent has a lower adsorption capacity than the CNF-GO aerogel adsorbent. The addition of GO will affect the adsorption capacity; the more GO is added, the higher the adsorption capacity.

### 2.5. Cr(VI) Adsorption

Adsorption testing of the CNF aerogel and CNF-GO aerogel was carried out by immersing them in a Cr(VI) solution with 50 ppm and 100 ppm concentrations. The initial aim of the study was to evaluate the adsorption performance of CNF-based aerogels at two concentration ranges around 50 ppm (low concentration) and 100 ppm (high concentration) [52]. The measured concentrations of 103.62 ppm, 99.79 ppm, and 46.87 ppm were the resulting working concentrations after preparing the Cr(VI) solutions and calibrating for experimental accuracy. These values closely align with the intended target ranges. The effect of contact time on the adsorption of Cr(VI) varied between 15 and 30 min at around 100 ppm and at 15 min for around 50 ppm [53].

**Figure 10:**
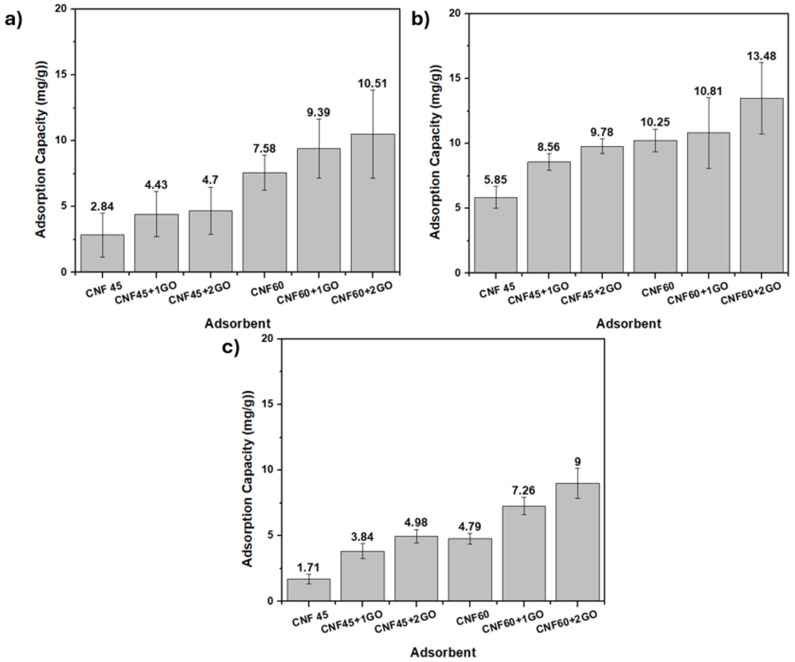
(**a**) Histogram of AAS analysis result in Cr(VI) solution with a concentration of 103.62 ppm for 15 min, (**b**) 99.79 ppm for 30 min, (**c**) 46.87 ppm for 15 min.

AAS analysis of the Cr(VI) solution with a control of 100 ppm Cr(VI) metal yielded a concentration of 103.62 ppm. The results of the AAS testing for this solution with a 15 min adsorption contact time can be seen in Figure 10a. The adsorption capacity of the CNF 60 aerogel adsorbents was better than that of the CNF 45 aerogel adsorbents. Additionally, adding GO to the CNF 45 and CNF 60 aerogel adsorbents enhanced their adsorption capacity. In the CNF 45 aerogel adsorbents, there was an increase in adsorption capacity of 2 mg/g, while in the CNF 60 aerogel adsorbents, there was an increase of 3 mg/g. The adsorption capacity of the aerogel adsorbents for Cr(VI) metal was lower compared to for Pb(II) metal.

This is because Pb(II) metal has a larger ionic radius than Cr(VI) metal. Metal ions with a larger ionic radius are easier to absorb. In addition, Pb(II) metal has a lower charge than Cr(VI) metal, so it is easier to remove from the solution [60]. Due to its larger size and lower charge, Pb(II) metal has a lower charge density compared to Cr(VI) metal. Lower-charge-density ions tend to form stronger complexes with soft ligands like hydroxyl and carboxyl groups. In addition, Pb(II) has a lower electronegativity than chromium, making Pb(II) metal more likely to form covalent-like interactions with electron-rich groups like hydroxyls and carboxyl.

Subsequently, the results of the AAS analysis for the Cr(VI) solution with a longer adsorption contact time, namely 30 min, and a control concentration of 99.79 ppm Cr(VI) are shown in Figure 10b. Compared to the 15 min contact time, the 30 min contact time shows a higher adsorption capacity for CNF aerogel adsorbents and CNF-GO aerogel adsorbents. The adsorption capacity for the CNF 45 aerogel adsorbents exhibits an increase of 5 mg/g, while the CNF 60 aerogel adsorbents show a rise of 4 mg/g. The optimum adsorption capacity is achieved by the CNF 60 aerogel adsorbents with the addition of 2 mL of GO, reaching 13.48 mg/g.

The AAS analysis results of the Cr(VI) solution with a control of 50 ppm Cr(VI) metal and a concentration of 46.87 ppm were obtained with a 15 min adsorption contact time, as shown in Figure 10c. In the Cr(VI) solution with a concentration of 46.87 ppm at a 15 min contact time, the same trend as for the 103.62 ppm concentration at 15 min was observed. That is, the CNF-GO aerogel adsorbents exhibited a better adsorption capacity than the CNF aerogel adsorbents, and the amount of adsorption increased with the increasing volume of added GO. GO can increase adsorption capacity because more oxygen-containing functional groups are available to interact with contaminants, more pores and channels exist within the aerogel structure, and it provides an even greater specific surface area for adsorption [58]. The optimum adsorption capacity was achieved with the CNF 60 aerogel adsorbents with the addition of 2 mL of GO, reaching 13.48 mg/g.

The results of this study show that the adsorption capacity of the CNF-GO aerogel is higher than that of Zhang et al. [61], who produced adsorbents from a citric acid-incorporated CNF with an adsorption capacity of only 10 mg/g at a contact time of 60 min and a Cr(VI) ion concentration of 70 ppm. The high adsorption capacity of CNF-GO is due to the high density of oxygen-containing functional groups, such as hydroxyl, carboxyl, and epoxy, which are hydrophilic and can form strong hydrogen bonds with water molecules. These groups increase the surface area and adsorption capacity. In addition, the relatively large spacing between the layers in GO allows water molecules to easily permeate and diffuse along the nano-capillaries, thus providing more pathways for water molecules to adsorb and increasing the adsorption capacity.

## 3. Conclusions

In this work, cellulose was successfully extracted from banana stem sheaths through a process of alkali hydrolysis and bleaching, and cellulose nanofiber was isolated using an ultrasonicator with an average diameter size of 14.6 nm for CNF 60 and 44.16 nm for CNF 45. The FTIR results confirmed the formation of CNF with the disappearance of lignin and hemicellulose peaks at 1311.4 cm^−1^ and 1596.7 cm^−1^ in the spectrum, and XRD showed characteristic CNF features, with high peaks at an angle of 2θ around 22.16°. The CNF/GO aerogel was obtained by using the freeze-drying technique, with a porous structure, low density, and high porosity. The fabrication was successfully carried out with compositions of CNF and GO, namely CNF 45 aerogel adsorbent, CNF 60 aerogel adsorbent, CNF45/GO1 aerogel adsorbent, CNF45/GO2 aerogel adsorbent, CNF60/GO1 aerogel adsorbent, and CNF60/GO2 aerogel adsorbent. The SEM images demonstrate that all the CNF and CNF-GO aerogel adsorbents were porous. The pore size diameter of CNF 60 aerogel adsorbents was smaller than that of CNF 45 aerogel adsorbents, with an average size of 109 µm. The adsorption capacity of the CNF and CNF-GO aerogel adsorbents was more effective for heavy metal lead ions than chromium. For lead Pb(II) adsorption, the optimum adsorption was achieved with CNF60/GO2 at a concentration of 100 ppm and a contact time of 30 min, reaching 27.27 mg/g. Meanwhile, the adsorption capacity for chromium Cr(VI) was obtained with CNF60/GO2 at a concentration of 100 ppm and a contact time of 30 min, reaching 13.48 mg/g. Thus, this research shows that cellulose nanofiber from banana stems can be used as an effective aerogel adsorbent to capture heavy metal ions, especially lead and chromium.

## 4. Materials and Methods

### 4.1. Materials and Chemicals

The banana peduncles from the Tanduk variety (*Musa paradisiaca*) were obtained from a local market in Bandung, West Java, Indonesia. NaOCl (12%) was purchased from Sakura Chemical, Bandung, Indonesia. Sodium hydroxide (NaOH) (98%), Pb(NO_3_)_2_, and K_2_Cr_2_O_7_ were purchased from Merck, Darmstadt, Germany. GO in the form of a colloid solution with a concentration of 10 mg/mL was obtained from Xfnano Materials Tech Co., Ltd., Nanjing, China.

### 4.2. Isolation of CNF from Banana Peduncle

Banana peduncles were cleaned under running water and then peeled bunches of skin were cut into round sheets with a thickness of approximately 1 cm. The peeled banana peduncles were then dried in an oven at 100 °C for 7 h. The dried banana peduncle was then ground using an FCT-Z200 grinder, Bandung, Indonesia, resulting in a brownish powder. The resulting powder was then meshed using a num-200 mesh sieve to obtain a homogeneous powder size.

The chemical treatment involving NaOH and NaOCl for the delignification and removal of hemicellulose and lignin from the banana peduncle was adapted from the study by Khawas and Deka, which extensively describes the process of isolating cellulose from culinary banana peel. The study emphasizes the effective removal of lignin and other impurities through sequential chemical treatments, including alkali and bleaching steps. This methodology has been widely used for cellulose isolation in various plant-based sources [62].

An amount of 125 g of banana peduncle powder was dissolved in 1000 mL of NaOH solution (1:8 ratio) for 4 h at 80 °C under stirring, using a magnetic stirrer. Afterwards, the mixture was filtered and washed until the filtrate was pH neutral [62]. The solid product was bleached twice using NaOCl solution at 60 °C, with a 4% concentration in the first 30 min cycle and a 2.5% concentration in the second. The resulting cellulose was dried in an oven for 24 h at 60 °C and then crushed to obtain pure cellulose powder. CNF was obtained using the ultrasonication method with two different time parameters: 45 min for CNF 45 and 60 min for CNF 60. A 2.5 g cellulose powder was dispersed in 100 mL distilled water. The mixture was ultrasonicated using an ultrasonic homogenizer JY96-IIN with power at 60% with 1 s on and off intervals for 45 or 60 min, resulting in a white CNF gel product.

### 4.3. CNF and CNF/GO Aerogel Adsorbent Fabrication

The aerogel adsorbents in this study are made of CNF (CNF 45 and CNF 60) and CNF modified with GO (CNF/GO). The GO addition parameter variation is 1 and 2 mL for every 4 mL CNF. The mixture of CNF and GO was stirred using a magnetic stirrer for 1 h, and then the CNF/GO mixture was ultrasonicated for 30 min to obtain a homogeneous mixture. The CNF and CNF-GO solutions were poured into a cylindrical mold (17 mm x 15 mm) and were stored overnight in a refrigerator at 4 °C for pre-cooling to avoid macroscopic cracks during the freeze-drying stage. Then, the samples were freeze-dried for 48 h at −40 °C to obtain the CNF and CNF/GO aerogel adsorbents [45].

### 4.4. Characterization

An X-ray diffraction (XRD) test used a Bruker D8 Advanced, Karlsruhe, Germany, to observe the diffraction pattern and calculate the degree of crystallinity. Diffraction patterns were obtained using a CuKα radiation source (λ = 0.15406 nm) at an angle range of 10–80° (2θ), with a set current and voltage of 40 mA and 40 kV, respectively. Calculating the degree of crystallinity (Ic) from the diffraction pattern involved area-based methods. The peak area-based method compares the crystal area and amorphous area via fitting deconvolution separation using a Gaussian function on Equation (1). Fitting deconvolution using a Gauss function was performed to confirm the amorphous region.

(1) $$I_{c}=\frac{A_{crystalline}}{A_{crystalline+amorphous}}\times 100\%$$

where A_crystalline_ is the total integral of the crystalline area, and A_crystalline+amorphous_ is the complete integration of all splitting peaks, including amorphous [63,64].

The Fourier-transform infrared spectroscopy (FTIR) test was carried out using Bruker Alpha II, Germany. The spectra were measured at a resolution of 4 cm^−1^ with a number of 50 scans, at a wavenumber of 500–4500 cm^−1^. A test was performed to identify the functional groups of GO, banana peduncle powder, cellulose, and cellulose nanofiber. Samples were tested using the attenuated total reflectance (ATR) technique.

To determine their size and morphology, transmission electron microscopy (TEM) was carried out by a Hitachi HT7700/TEM, Tokyo, Japan on CNF samples. Before measurements were taken, the samples were coated with gold. The AAS test was performed using Shimadzu AA-6300. AAS was carried out to determine the concentration of Pb(II) solution and Cr(VI) solution before and after adsorption.

A scanning electron microscopy (SEM) test is performed to obtain the surface morphology, pore shape, and cross-section morphology of the CNF, CNF aerogel adsorbent, and CNF/GO aerogel adsorbent. The SEM test is carried out using JEOL-IT300, JEOL Ltd., Tokyo, Japan. The sample was coated with gold (Au) before testing. In addition, an EDS mapping test is also performed on aerogel adsorbent samples that were immersed to determine the distribution of adsorbed lead on the surface.

### 4.5. Porosity

The porosity of the CNF and CNF/GO aerogel adsorbents was determined using an immersion method in n-butanol. Each sample was immersed in an n-butanol solution for 2 h. The weight of the aerogel adsorbent before and after immersion was recorded and calculated based on Equation (2) [65].

(2) $$Porosity\left(\%\right)=\frac{MBuOH/\rho BuOH}{\frac{MBuOH}{\rho BuOH}+MS/\rho S}$$

MBuOH = weight of butanol absorbed (g);

ρBuOH = butanol density (g/mL);

MS = weight of samples (g);

ρS = samples density (g/mL).

### 4.6. Adsorption Experiment

The adsorption test was carried out by adding aerogel adsorbents (175 mm × 145 mm) weighing 0.0800–0.1 g into 30 mL of Pb(II) for an ion test using a Pb(NO_3_)_2_ solution with 100 and 50 ppm concentrations while stirring at 100 rpm. The samples were weighed before and after immersion. Immersion was performed by varying the adsorption contact time, 15 and 30 min. The same treatment was also applied in the Cr(VI) ion test using a K_2_Cr_2_O_7_ solution. Then, the samples were analyzed using AAS Shimadzu AA-6300, and an average decrease in the metal ion levels was observed for each sample. The heavy metal rates before and after treatment were then compared to determine the heavy metal reduction levels. The capacity adsorption of the aerogel adsorbent was calculated based on Equation (3) [66].

(3) $$q_{e}=\frac{(C_{0}-C_{e})\times V}{m}$$

where

q_e_ = adsorption capacity (mg/g);

C_0_ = initial concentration of heavy metal in solution (mg/L);

C_e_ = equilibrium concentration of heavy metal in solution (mg/L);

V = volume of the solution (L);

m = mass of the adsorbent (g).

### 4.7. Micro-CT

Micro-CT was carried out by using a micro-CT (Scanner Skyscan 1173, Bruker, Kontich, Belgium) High-Energy device with high resolution scanning at 8.57 um pixels. Micro-CT was performed on the CNF aerogel adsorbent and CNF/GO aerogel adsorbent to obtain the porosity values and discern the 3D architectures. A total of 1053 slice images for the CNF 60 aerogel adsorbents and 856 slice images for the CNF 60+2 mL GO aerogel adsorbents were generated from the micro-CT. Each layer’s 2D image was constructed using Nrecon software (NRecon 1.7.5.0, Bruker) and later reconstructed with CTvox software (by Skyscan, Bruker, version 3.3.0). Finally, the data were processed with Abaqus software version 2023HF2 to simulate compression along the z-axis. The relationship between thickness and Young’s Modulus was analyzed to determine the material’s hyper-elastic and viscoelastic properties.

## Figures and Tables

**Table 1:** Micro-CT test result.

Sample	Interconnected Pore (%)	Total Porosity (%)
CNF 60 aerogel adsorbents	92.4	92.4
CNF 60 + 2 mL GO aerogel adsorbents	91.4	91.41

## Data Availability

Data that support the findings of this study are available from the corresponding author upon reasonable request. Please contact us if you are interested in sharing the data.
